# Liver Transplantation for Alcoholics with Terminal Liver Disease

**Published:** 1996

**Authors:** David H. Van Thiel

**Affiliations:** David H. Van Thiel, M.D., is the medical director of transplantation at the Transplant Center, University of Kentucky Medical Center, Lexington, Kentucky

**Keywords:** liver, organ transplantation, alcoholic liver disorder, alcoholic liver cirrhosis, mortality, ethics, treatment complications, public policy, patient treatment matching, prognosis

## Abstract

Most candidates for liver transplantation have irreversible cirrhosis caused by years of heavy alcohol consumption. Arguments against liver transplantation for alcoholics include the presumption of relapse to heavy drinking, which might damage the new liver or lead to its rejection. Corresponding ethical arguments focus on the presumption that alcoholics brought their condition upon themselves and should not compete with nonalcoholics for scarce donor livers. However, experimental data demonstrate that carefully selected alcoholics can survive liver transplantation and return to the workplace as productive citizens. Moreover, it has never been considered ethical for clinicians to refuse treatment to patients for diseases that are partly or wholly preventable.

Alcoholic liver disease is one of the most serious medical consequences of long-term alcohol use. Moreover, long-term heavy alcohol use is the most prevalent single cause of illness and death from liver disease in the United States. More than 25,000 Americans died of liver cirrhosis in 1991, making it the eleventh most frequent cause of death that year (National Center for Health Statistics 1994). Approximately one-half of cirrhosis deaths have been attributable to alcohol consumption (see sidebar) (National Institute on Alcohol Abuse and Alcoholism [NIAAA] 1993).

**Definition of Terms**

The threshold level of alcohol consumption that causes cirrhosis in susceptible subjects is unknown. Generally speaking, alcoholic cirrhosis develops in susceptible subjects who have been consuming a daily average of a “fifth” of 80-proof vodka or the equivalent for 10 to 20 years (Senior et al. 1988; National Institute on Alcohol Abuse and Alcoholism 1993). Therefore, although standard diagnostic criteria for alcoholism do not include measures of quantity consumed, the general use of the term “alcoholism” in this article reflects the assumption that only alcoholics are likely to sustain the frequency and duration of drinking that leads to cirrhosis. This is a definition of convenience, because heavy drinkers—especially women—can get cirrhosis without meeting the criteria for alcoholism.

The term “alcohol abuse” as used here refers to any drinking that causes problems for the drinker or for others. In discussions of specific research, the terms are used as in the article cited.

ReferencesNational Institute on Alcohol Abuse and AlcoholismAlcohol and the Liver. Alcohol AlertNo. 19. PH 329Bethesda, MDNational Institutes of Health1993SeniorJRRedekerAGMaddreyWCGalambosJTRecognition and Management of Alcoholic HepatitisDHHS Pub. No. (ADM) 88–1547Washington, DCU.S. Govt. Print. Off1988

The only effective treatment for patients whose liver disease (usually cirrhosis) has become terminal and irreversible is transplantation. Because most cases of terminal liver disease are related to heavy alcohol consumption (Senior et al. 1988), the majority of potential candidates for liver transplants are alcoholics. However, some transplant centers in the United States have been unwilling to provide the procedure to people with alcohol-induced liver injury (Kumar et al. 1990). This article explores the reasons for and against liver transplantation for patients with alcoholic liver disease and provides evidence suggesting that alcoholics should be eligible for this life-saving treatment.

## Normal Liver Function

The liver is the largest organ of the body, located in the upper right section of the abdomen. As well as being involved in many of the body’s metabolic systems, the liver assists in digesting, absorbing, and processing food. A versatile organ, the liver stores vitamins, synthesizes cholesterol, controls blood fluidity, and regulates blood-clotting mechanisms. It also filters circulating blood, removing and destroying toxic substances. Thus, liver disease compromises the body’s ability to perform multiple functions essential to life.

## Alcoholic Liver Disease

Alcohol-related liver damage includes fatty liver, alcoholic hepatitis, and cirrhosis. A single episode of heavy drinking is sufficient to cause some deposition of fat in the liver, which, however, rarely causes illness (NIAAA 1993). Long-term heavy drinking may lead to alcoholic hepatitis, a severe inflammation of the liver characterized by nausea, weakness, pain, loss of appetite, weight loss, and fever (Senior et al. 1988). Alcoholic cirrhosis is the most advanced form of alcoholic liver injury, characterized by progressive development of scar tissue that constricts blood vessels and distorts the liver’s internal structure, impairing liver function. Approximately 10 to 20 percent of heavy drinkers develop cirrhosis (NIAAA 1993).

A patient may have only one of these three conditions or any combination of them. Traditionally, alcoholic liver disease has been conceptualized as progressing from fatty liver to alcoholic hepatitis to cirrhosis. However, cirrhosis may appear insidiously, without any previous stage resembling hepatitis, and alcoholic hepatitis can be fatal by itself without leading to cirrhosis (Senior et al. 1988)

Fatty liver rarely requires treatment, and alcoholic hepatitis is generally reversible upon abstinence. Cirrhosis initially is reversible, but past a certain point, progression is relentless and only replacement with a healthy liver can save the patient’s life (NIAAA 1993). Thus, transplantation is the only cure for advanced alcoholic cirrhosis.

## Constraints on Liver Transplantation

Liver transplantation is a traumatic and costly procedure. Total hospital time for surgical recovery averages 4 to 5 weeks; during this time, the patient begins taking immunosuppressive medications to discourage rejection of the transplanted liver. Following hospital discharge, the patient may attend a clinic as often as once per week for up to 6 months for further medical management until the patient’s condition can be judged stable and the transplant successful (Merion 1994). Costs are substantial, ranging from several hundred thousand dollars to well over $1 million. In general, these costs are borne by national and private insurance.

The scarcity of livers suitable for transplantation affects alcoholics and nonalcoholics alike. Approximately 3,000 human livers are transplanted in the United States each year (Beresford 1994*b*), distributed among as many as 64,000 potential applicants (Moss and Siegler 1991).

Transplant teams decide which patients qualify as recipients for scarce donor livers and thus have a responsibility to consider whether the candidates have a good chance not only of surviving the transplant but of subsequently leading healthy and productive lives. The selection procedure involves not only medical judgments about a patient’s physical and mental health but social and community concerns as well.

## Arguments Against Liver Transplants for Alcoholics

Arguments for evaluating alcoholics differently from non-alcoholics as recipients for liver transplants have been based on both medical and ethical grounds.

### Medical Grounds

Alcoholics historically have been considered unsuitable for liver transplantation because of their presumed high risk of relapse to excessive drinking after transplantation. Posttransplant alcohol abuse may lead to noncompliance with the complex regimen of immunosuppressive therapy and other crucial posttransplant management, with subsequent rejection of the new liver (Lucey 1994). Moreover, a return to former drinking habits may damage the new liver, ruining a precious donor organ that might have saved another person’s life. In addition, patients who have consumed alcohol heavily enough to develop terminal liver disease are likely to have damaged other organs in addition to their livers, further endangering their prospects of a successful recovery following transplantation. Thus, placing rare livers in these patients could be risky and, at worst, could waste the donor organ (Kumar et al. 1990; Gasbarrini et al. 1992).

### Ethical and Public Policy Grounds

Donor livers are an extremely scarce and nonrenewable resource. Because alcohol consumption is responsible for approximately one-half of all cases of terminal liver disease, the inclusion of even carefully selected alcoholics in the pool of potential liver recipients is likely to place considerable strain on the limited supply of transplantable livers. If allowed to compete equally for access to transplantation, some alcoholic patients will certainly receive a liver before some nonalcoholic patients, who will subsequently die (Benjamin and Turcotte 1994).

Moss and Siegler (1991) address this issue, arguing that alcoholics have an obligation to prevent their alcoholism from progressing to the stage of organ damage; thus, fairness dictates that alcoholics should be given lower priority than patients whose liver disease was not preventable (Moss and Siegler 1991). Some authors have gone on to say that because alcoholism constitutes “self-abuse,” alcoholic liver damage merits less attention than do other forms of liver disease (Gasbarrini et al. 1992).

Moss and Siegler (1991) note further that because of the high cost of liver transplantation, public support is crucial to maintain insurance coverage in the current era of cost containment. Moreover, the scarcity of livers for transplantation reflects public reluctance to donate organs or lack of awareness of the importance of organ donation. Giving equal footing for transplants to people with alcoholic liver disease might erode public support for the costly procedure and ultimately result in fewer donated livers and a decline in insurance coverage (Moss and Siegler 1991).

## Arguments for Liver Transplants for Alcoholics

### Refuting Medical Grounds

Experimental data demonstrate that carefully selected alcoholics can survive liver transplantation and return to the workplace as productive citizens. For example, Poynard and colleagues (1994) found that liver transplantation increased the 2-year survival rates of patients with severe alcoholic cirrhosis compared with similar patients who had not received transplants.

Additional studies have examined the factors that contribute to a good prognosis among alcoholic transplant patients. For example, from a followup survey conducted between 6 months and 3 years after transplant operations, Beresford and colleagues (1992) found that 9 percent of their alcoholic patients had returned to drinking at a level symptomatic of alcoholism and 14 percent reported some alcohol consumption—a rather low rate of recidivism. By comparison, 46 percent of a nonalcoholic group of liver transplant patients reported drinking socially. Among other measures, the two groups differed little in their compliance with medications and their psychiatric morbidity following their operations. The researchers concluded that selected patients with alcoholic liver disease merit consideration for liver transplants (Beresford et al. 1992).

Similarly, Kumar and colleagues (1990) surveyed 52 surviving patients (of an original group of 73) who had received transplants an average of 25 months previously for alcoholic liver disease. The survival rate for the alcoholics did not differ from that of patients with nonalcoholic liver disease who had received transplants (excluding patients with hepatitis B or hepatic cancer, who have the poorest outcomes of all groups). Although six alcoholic patients (11.5 percent) returned to drinking, four drank only socially and two drank moderately. Significantly, 54 percent reported that they were employed (full time, part time, or self-employed), 21 percent were homemakers, and 21 percent were unable to work (two other patients were willing but unable to find work). This rate of employment was no different from that seen in groups of transplant survivors whose liver disease was unrelated to alcoholism (Kumar et al. 1990). Thus, alcoholic patients in this study appeared to have the same recovery success as did nonalcoholics after liver transplantation. Their rate of returning to abusive drinking was also low (Kumar et al. 1990).

Quality-of-life measurements are receiving increasing prominence in studies of treatment outcome. Carrington and colleagues (1996) surveyed alcoholic and nonalcoholic liver transplant recipients 1 to 3 years after their operations to compare measures of (among other factors) emotional and physical problems (e.g., anxiety, stress, or pain and discomfort), energy level, compliance with medical regimens, and quality of social relationships. The researchers found no significant differences between the groups in any of the areas surveyed (Carrington et al. 1996).

Reviewing the success of patients receiving liver transplants for a combination of alcoholic hepatitis and cirrhosis, Bonet and colleagues (1993) found that compared with patients with alcoholic cirrhosis alone, alcoholic hepatitis patients had a similar short-term survival rate after transplantation. The authors noted that hepatitis patients tended to receive transplants under more urgent conditions than did cirrhotic patients because of more rapid progression of liver failure in alcoholic hepatitis. Thus, many hepatitis patients had not maintained abstinence from alcohol for a period of months before the operation, unless they had already been hospitalized. This was reflected in the finding that 89 percent of the cirrhosis patients remained sober after their liver transplant operations compared with only 51 percent of those who received transplants for alcoholic hepatitis (Bonet et al. 1993).

### Refuting Ethical and Public Policy Grounds

Refuting the argument that alcoholic patients have a personal responsibility to prevent their own liver disease and therefore should not be considered on an equal basis with other liver transplant candidates, Benjamin and Turcotte (1994) contend that rules not applied to other “lifestyle-related” diseases—such as lung disease from smoking or cardiovascular disease from chronic obesity—should not be applied to alcoholism, regardless of the scarcity of the treatment in question. In addition, these scientists suggest that the most responsible use of a donor organ may, in many cases, involve giving it to an alcoholic patient who is a better match (i.e., whose blood type matches that of the donor) and who needs it urgently rather than to a non-alcoholic patient who is a bad match and whose need is less urgent. The latter scenario could occur if Moss and Siegler’s (1991) rules for preferential treatment were applied to liver transplant candidates.

According to Benjamin and Turcotte (1994), to maintain public support for expensive liver transplantation procedures by discriminating against alcoholics is to base public policy on distorted public perceptions. For example, the public perception of alcoholism is strongly influenced by the image of the “drunk driver” and the tragedy wrought by alcohol-impaired drivers (Benjamin and Turcotte 1994). However, not all drunk drivers are alcoholics, and not all alcoholics drive while impaired. Sufficient evidence indicates that alcoholism is a disease and that a large part of susceptibility to this disease is genetic and beyond a person’s control. An alcoholic’s failure to control his or her drinking problem does not represent moral weakness, especially in a society that glamorizes alcohol consumption. Benjamin and Turcotte (1994) conclude that a sound, fact-based liver transplantation policy can be adequately defended to the public.

## Criteria for Selecting Alcoholic Patients for Liver Transplantation

Studies support the idea that the key to successful liver transplantation is careful selection of recipients. Beresford and colleagues (1990, 1992) evaluated patients with liver disease before they became candidates for liver transplantation. The researchers assessed whether patients were alcohol dependent and, if so, whether they recognized their alcoholism. The researchers also assessed the patients’ psychiatric stability and other factors that might affect their prognosis, such as the presence of supportive social relationships and activities that could substitute for drinking in their lives. These factors appear to be associated with alcoholics’ ability to remain abstinent independent of transplantation and thus can serve as a starting point for predicting a patient’s prognosis following a transplant procedure (Beresford et al. 1990, 1992). (Additional studies from other transplant centers in the United States further support these findings; for reviews, see Beresford 1994*a*,*b*; Beresford and Lucey 1994.) Van Thiel and colleagues (1991) have gleaned from their own and other liver transplantation studies (e.g., Beresford et al. 1990) several criteria that predict successful recovery:

- The support of a significant other
- The patient’s acceptance of alcoholism as the cause of his or her liver disease
- A job—or adequate training or education to obtain employment—and the redirection of the patient’s interests away from drinking to other pursuits.

Beresford and colleagues (1992) also support the careful selection of alcoholics for transplant candidacy. Like Van Thiel and colleagues, Beresford (1994*a*) suggests that the support of a significant other, the patient’s recognition of his or her alcoholism, and the presence of social supports and alternative pursuits are important to a successful outcome. He also adds, however, that a sense of hope for the future is crucial. A positive attitude may be engendered by maintaining patient interactions with the transplant team during early recovery and by patient participation in a rewarding activity (Beresford 1994*a*,*b*). Beresford and colleagues (1992) argue for selecting alcoholics for transplant candidacy on a case-by-case basis, rather than relying solely on general criteria. Thus, if an alcoholic patient has a good prognosis for recovery, abstinence, and subsequent participation in society, he or she should not be denied transplant candidacy for failing a criterion, such as a specific period of abstinence before the transplant procedure. Finally, Beresford and colleagues (1992) suggest that long-term followup of alcoholic transplant recipients should further clarify the factors that best predict long-term abstinence from alcohol (which contributes to a successful outcome) after surgery.

## Conclusions

The history of Western medicine is totally inconsistent with the concept of denying treatment to people because their behavior led to their illness. On the contrary, practitioners are bound to provide care to people who show signs of ill health, regardless of the cause. The behavior leading to alcoholic liver disease is itself the result of a disease—alcoholism. Why, then, does disagreement exist over whether alcoholics with liver disease should be considered equally with nonalcoholic patients for rare donor livers, if the alcoholics are equally likely to recover and reenter society as healthy, productive citizens?

The unique qualities of liver transplantation include the scarcity of the resource (i.e., the organs) and the need for public support for the procedure. Multiple factors influence the development of alcoholism, the recognition that one has alcoholism, the knowledge that alcoholism is a treatable illness, and the decision to seek treatment. Therefore, science cannot assess the extent to which alcoholics can be held personally responsible for becoming alcoholic or for seeking their own alcoholism treatment. Moreover, it is difficult to determine in all cases whether cirrhosis has been caused by heavy drinking or by other factors (Benjamin and Turcotte 1994). Therefore, selection for liver transplantation should be made with regard to medical necessity and the potential for a successful outcome independent of diagnosis or cause (Beresford and Lucey 1994). This should apply to those with alcoholic liver disease as it does for all other forms of liver disease.^1^

## Footnotes

1 *Editor’s note:* In November 1996 the popular press erroneously implied that revised transplantation guidelines had decreased the eligibility of alcoholics for liver transplants. In actuality, the medical criteria for classifying urgency of need had been clarified. These changes do not affect an alcoholic patient’s chances of receiving a liver.
